# Dynamic changes in lactate-related genes in microglia and their role in immune cell interactions after ischemic stroke

**DOI:** 10.1515/med-2025-1178

**Published:** 2025-04-15

**Authors:** Jinzhong Yao, Huan Deng, Peng Wang, Bo Li, Zaisheng Qin

**Affiliations:** Department of Anesthesiology, Nanfang Hospital, Southern Medical University, Guangzhou 510000, China; Department of Anesthesiology, The Eighth Affiliated Hospital of Sun Yat-sen University, Shenzhen, 518033, China; Department of Orthopedics, Beijing Luhe Hospital, Capital Medical University, Beijing, 101101, China

**Keywords:** ischemic stroke, microglia, single-cell RNA sequencing, lactate metabolism

## Abstract

**Objectives:**

This study aims to elucidate the dynamic changes in lactate-related genes (LRGs) in microglia following ischemic stroke (IS) and their associations with immune cells.

**Methods:**

We performed differential expression analysis on bulk-sequencing (GSE30655 and GSE35338) and scRNA-seq data (GSE174574) to identify differentially expressed genes. These genes were intersected with lactate genes from MSigDB to identify post-stroke LRGs. We used t-SNE to visualize LRG distribution across cell types and selected microglia for cell–cell communication, pseudo time, and functional enrichment analyses. These findings were integrated with the GSE225948 scRNA-seq dataset to examine LRG trends in the chronic phase of IS. Finally, CIBERSORT was used to explore immune cell infiltration changes and LRG-immune cell associations post-IS.

**Results:**

Nine LRGs were identified, including Spp1, Per2, Col4a1, Sfxn4, C1qbp, Myc, Apln, Cdo1, and Cav1, with Spp1, C1qbp, and Myc highly expressed in microglia. C1qbp and Myc are crucial in the acute phase, while Spp1 impacts both acute and chronic phases of IS. Microglia subcluster analysis revealed four subclusters (MG0-MG3). Immune cell infiltration analysis showed significant associations between these genes and immune cells.

**Conclusion:**

In summary, Spp1, C1qbp, and Myc are LRGs that are predominantly expressed in microglia and play regulatory roles in various stages of IS.

## 1 Introduction

Ischemic stroke (IS) is a prevalent and devastating condition that leads to significant disability and poses a substantial health burden, with many survivors facing long-term impairments and diminished quality of life [1]. Among the various types of strokes, IS constitutes approximately 80% and is caused by sudden blockage of the cerebral artery, often due to a blood clot or thrombus [2]. This blockage leads to ischemic damage, where the affected brain tissue does not receive adequate blood supply, causing cell death and permanent neurological impairment [3].

Microglia are crucial for the initiation and perpetuation of cerebral infarction through their adoption of distinct polarization states following ischemia, thereby exerting a substantial impact on the neuroinflammatory response and subsequent neuronal damage [4]. Following ischemia, microglia rapidly polarize into two distinct states: the pro-inflammatory M1 phenotype and the anti-inflammatory M2 phenotype [5]. The polarization of microglia is governed by a complex interplay of factors such as peroxisome proliferator-activated receptor γ (PPARγ) [6], the interferon regulatory factor (IRF) family [7], and Toll-like receptor 4 (TLR4) [8], which are key regulators of microglial polarization.

During cerebral ischemic acidosis, substantial lactate accumulation occurs intracellularly as the primary mode of energy metabolism shifts from aerobic to glycolytic processes to maintain ATP supply [9]. Furthermore, the permeation of lactate from peripheral blood into the brain, facilitated by a compromised blood–brain barrier, exacerbates lactate accumulation within the brain [10]. Lactate, once viewed solely as a metabolic byproduct, is now recognized for its pivotal role in cellular function and its potential as a therapeutic target, supported by recent research into lactate metabolism and its disease treatment implications [11]. The role of lactate in modulating cellular function is further underscored by its link to synaptic plasticity, a key area of investigation in central nervous system (CNS) pathologies [12]. Additionally, lactate serves as a precursor that promotes histone lactylation and influences histone lysine lactylation (Kla) levels [13]. Lactylation of histone lysine residues, a novel post-translational modification (PTM), can stimulate gene transcription within chromatin, enhance the expression of homeostatic genes like arginase 1 (Arg1), and induce a phenotypic shift in macrophages from M1 to M2 [14]. Recent single-cell RNA sequencing (scRNA-seq) analyses have demonstrated that microglia are capable of rapid metabolic reprogramming and the use of various bioenergetic substrates, suggesting a regulatory role for lactate in microglial function [15]. Moreover, the research underscores the influence of lactate on microglial function, potentially harnessed to modulate neuroinflammation and enhance brain health, a process that may be attributed to the reprogramming of microglial glycolysis [15,16]. In mice and microglia, the H3K9 lactylation site is a key site involved in histone lactylation, which further promotes glycolysis and induces neuronal injury [17]. Furthermore, lactate can modulate the microglia inflammatory responses and alleviate cerebral ischemia injury by inhibiting the CCL7/NF-κB signaling pathway induced by HIF-1α [18]. Overall, it is evident that lactate accumulation due to ischemia can impact the biological functions of microglia. Identifying the LRGs in microglia post-IS will aid in understanding which LRGs are involved in the reprogramming process of microglia.

The advent of scRNA-seq has marked a significant paradigm shift in our understanding of cellular heterogeneity and gene expression dynamics, particularly in the context of IS [19]. This technology enables detailed profiling of gene expression patterns at the single-cell level, elucidating cellular response diversity, identifying rare cell types, and revealing complex transcriptional programs that underpin biological processes [20]. Most importantly, scRNA-seq offers unique insights into cell subpopulations and their functions in pathophysiological processes, particularly in the context of IS [21].

The primary objective of this study was to identify LRGs by integrating bulk sequencing and scRNA-seq analyses. Furthermore, this study aimed to elucidate the role of LRGs in modulating microglial responses to IS and identify potential gene targets for manipulation to enhance neuroprotection or mitigate ischemic injury. This approach could pave the way for the development of innovative therapies that target metabolic pathways in microglia, potentially reducing the severity of ischemic injury and promoting neurological recovery in patients with stroke.

## 2 Materials and methods

### 2.1 Data acquisition

**Figure 1:**
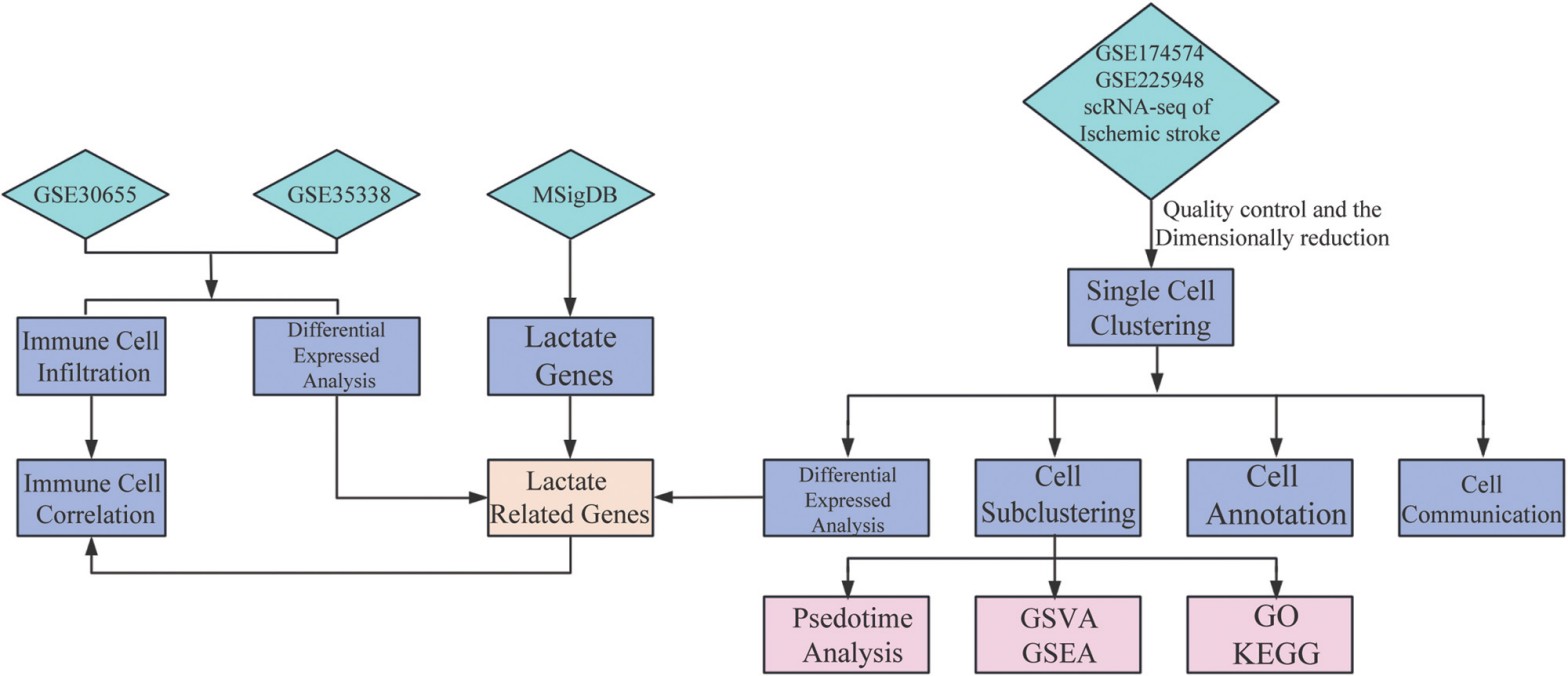
Flowchart of this study.

The research flow diagram is presented in Figure 1. The datasets utilized for our analysis were procured from the Gene Expression Omnibus (GEO) repository [22]. This public database archives and distributes high-throughput gene expression data. Specifically, we accessed two Series Matrix Files, GSE30655 and GSE35338, which were instrumental in our comparative genomic studies. Additionally, to facilitate the scRNA-seq analysis, we employed two single-cell data files identified as GSE174574 [23] and GSE225948 [24]. The overall description of these datasets is listed in Table 1. A total of 318 lactate-related genes (LRGs) were obtained from the Molecular Signatures Database [25] (http://www.gsea-msigdb.org/gsea/index.jsp).

**Table 1:** Detailed information of the datasets used in this study

GEO datasets	Platform	Sample source	Stroke cases	Control cases	Cohort type
GSE30655	GPL1261	Brain (*Mus musculus*)	7	3	Bulk RNA sequencing
GSE35338	GPL1261	Brain (*Mus musculus*)	5	4	Bulk RNA sequencing
GSE174574	GPL21103	Brain (*Mus musculus*)	3	3	Single-cell RNA sequencing
GSE225948	GPL9057	Brain (*Mus musculus*)	4	4	Single-cell RNA sequencing

### 2.2 Data integration and batch effect correction

Normalization of the datasets GSE30655 and GSE35228 was conducted via the “limma” R package [26] to merge into a unified, comprehensive dataset. To address potential batch effects, which could introduce bias into our analysis, we employed the Combat method facilitated by the “sva” R package [27]. This method was specifically designed to adjust for unwanted variations owing to batch effects, thus enhancing the accuracy and validity of our findings.

### 2.3 Differential expression analysis and visualization

Differential expression analysis was applied to identify genes with significant changes in expression between stroke groups and sham groups using the “limma” package in R [26]. Genes were considered significantly differentially expressed if they exhibited an absolute log2 fold change (|logFC|) greater than 0.5 and an adjusted *p*-value less than 0.05. The results were visualized through volcano plot via “ggplot2” and heatmap via “pheatmap” R package, respectively.

### 2.4 Single-cell data processing and cell annotation

We utilized the GEO datasets GSE174574 and GSE225948 for single-cell RNA sequencing analysis. Following rigorous quality control to exclude low-quality cells and those with excessive mitochondrial DNA content, we implemented the “LogNormalize” method to normalize gene expression values and stabilize variance across the dataset. The “vst” method in Seurat was employed to identify highly variable genes, which were subsequently subjected to principal component analysis for dimensionality reduction and to elucidate major sources of variation. Clustering was conducted based on transcriptomic profiles, and batch effects were mitigated utilizing the harmony algorithm. The “FindAllMarkers” function in Seurat, corroborated with the previous published literature, facilitated the annotation of cell types within each cluster. Finally, t-SNE visualization offers a comprehensive two-dimensional representation of the cellular landscape, highlighting distinct cellular populations.

### 2.5 Ligand–receptor interaction analysis (CellChat)

The CellChat R package was utilized to analyze ligand–receptor interactions based on normalized gene expression profiles, which facilitated the quantification of communication strengths between distinct cell types and the identification of key cellular communicators. The constructed cell–cell communication network was visualized through various graphical representations to illustrate the interactions and their intensities, thereby providing insights into potential signaling pathways.

### 2.6 Microglial subtype identification

To refine the classification of microglial subtypes and track the expression patterns of Spp1, C1qbp, and Myc, we analyzed their distribution across subtypes and compared their expression levels between stroke groups and sham groups. The spatial distribution of these genes was visualized using t-SNE/UMAP, and violin plots were used to represent their expression profiles.

### 2.7 Gene set variation analysis (GSVA)

GSVA is a non-parametric, unsupervised method employed to evaluate gene set enrichment dynamics across samples within an expression dataset. For this analysis, genesets were extracted from the Molecular Signatures Database (MSigDB) database using the “msigdbr” R package. Subsequently, lactate-related genesets were identified by filtering the comprehensive gene set with the keyword “lactate.” All four microglial subclusters were analyzed using LRGs to elucidate the biological function of each microglial type in lactate metabolism.

### 2.8 Gene set enrichment analysis (GSEA) pathway enrichment analysis

To elucidate the biological functions of Spp1, C1qbp, and Myc, we utilized bulk sequencing data for GSEA. Initially, we stratified the expression matrix into high- and low-expression groups based on the variation in the expression levels of these three genes and subsequently performed differential analysis using the limma package. Following ENTREZID conversion, we conducted an analysis using the clusterProfiler package and ultimately selected the top five pathways by normalized enrichment score for visualization.

### 2.9 Functional enrichment analysis of microglia MG1

To explore the biological functions and pathways of microglia MG1, we performed a functional enrichment analysis of the top 50 differentially expressed genes (DEGs) from these cells. Utilizing the ClusterProfiler R package, we conducted a Gene Ontology (GO) analysis to identify significantly enriched GO terms, which were visualized as a bar plot where the height and color of each bar represent the enrichment scores and gene counts, respectively. For pathway analysis, we investigated the Kyoto Encyclopedia of Genes and Genomes (KEGG) pathways using the same package, and the results were depicted in a bubble chart with bubble sizes indicating the number of associated genes and color intensity reflecting the significance of enrichment. Additionally, we employed a chord diagram to visually map the interactions between key genes.

### 2.10 Pseudotime trajectory analysis

Pseudotime trajectory analysis was conducted via Monocle 2 (version 2.32) to explore the microglial subclusters and examine the expression dynamics of Spp1, C1qbp, and Myc within these subtypes across stroke and sham groups. Through pseudotime trajectory analysis, microglial cells were ordered along a developmental sequence from less mature to more mature states based on the expression of key marker genes. This analysis allowed us to track changes in the expression of Spp1, C1qbp, and Myc during cellular development, visualized through UMAP plots colored by pseudotime values.

### 2.11 Immune cell infiltration analysis

To study the disease immune microenvironment, we used the R package “CIBERSORT” to calculate immune cell infiltration based on the merged bulk-seq dataset. The results were visualized in stacked bar plots to illustrate the distribution of immune cells across samples and box plots to assess their variability. Spearman correlation coefficients were calculated to measure LRGs and immune cell correlations, and the results were visualized using a correlation heatmap. Finally, correlation lollipop chart were used to specifically analyze the associations between LRGs and 22 immune cells.

## 3 Results

### 3.1 Identification of DEGs in stroke mice

**Figure 2:**
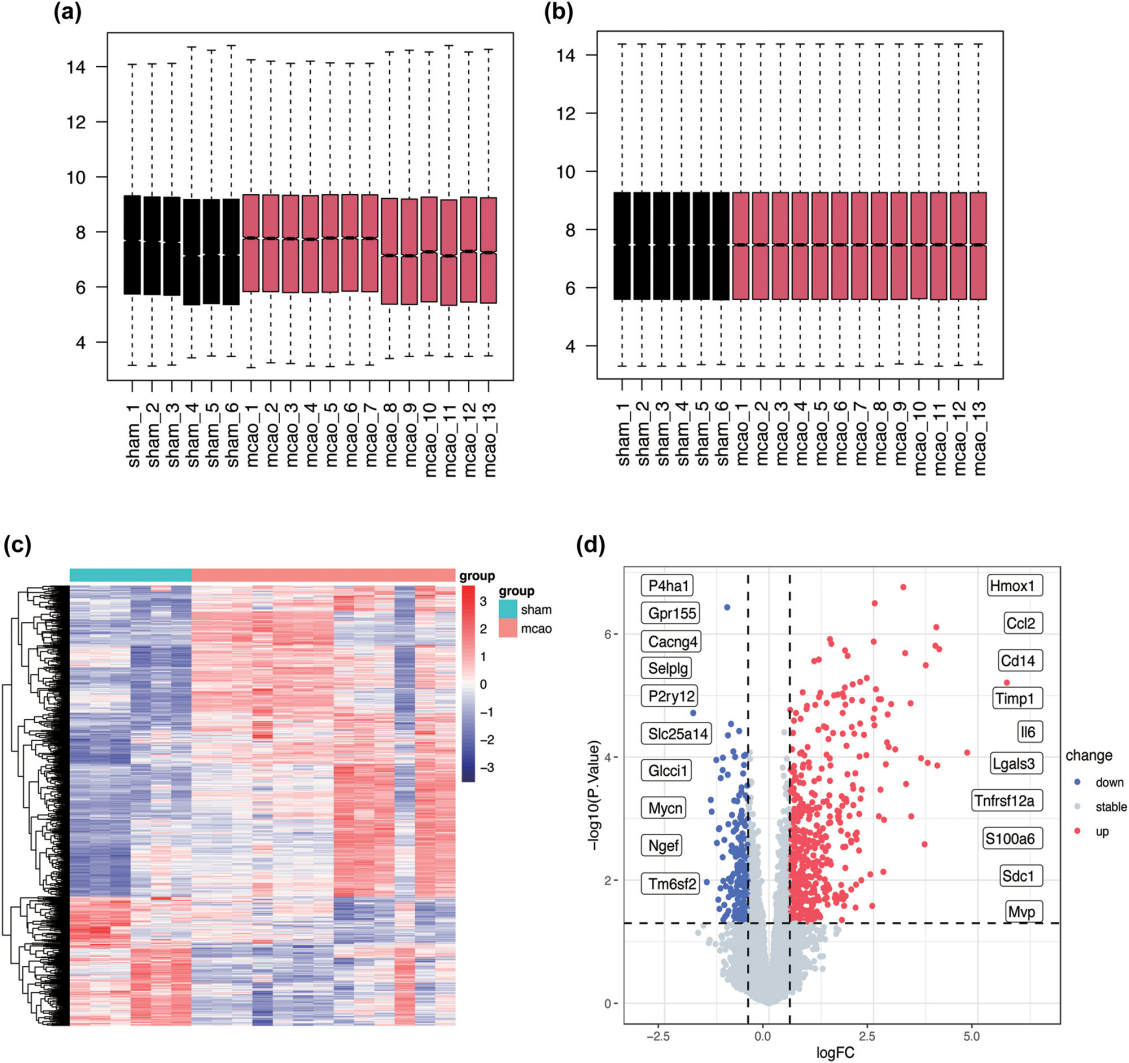
Datasets normalization and differential analysis (a) and (b) Comparison of GSE30655 and GSE35388 before and after batch effect correction. (c) Heatmap of the DEGs between sham and MCAO samples; red represents up-regulated genes, and blue represents down-regulated genes. (d) The volcano plot illustrates the distribution of DEGs, with the top 10 significantly up-regulated (blue) and down-regulated (red) genes marked for emphasis.

Box-plot analysis of the raw data demonstrated that the gene expression levels displayed heterogeneity across samples within both datasets (Figure 2a). This batch effect was subsequently mitigated through quantile normalization to ensure a more accurate comparison (Figure 2b). Using the selection criteria of |log2FC| > 0.5 and adjusted *p*-value <0.05, 682 DEGs were identified, comprising 482 upregulated and 200 downregulated genes (Figure 2c). The top ten upregulated and downregulated genes were selected for visualization using a volcano plot (Figure 2d).

### 3.2 Cell subpopulation annotation of single-cell data and lactate-related DEG identification

**Figure 3:**
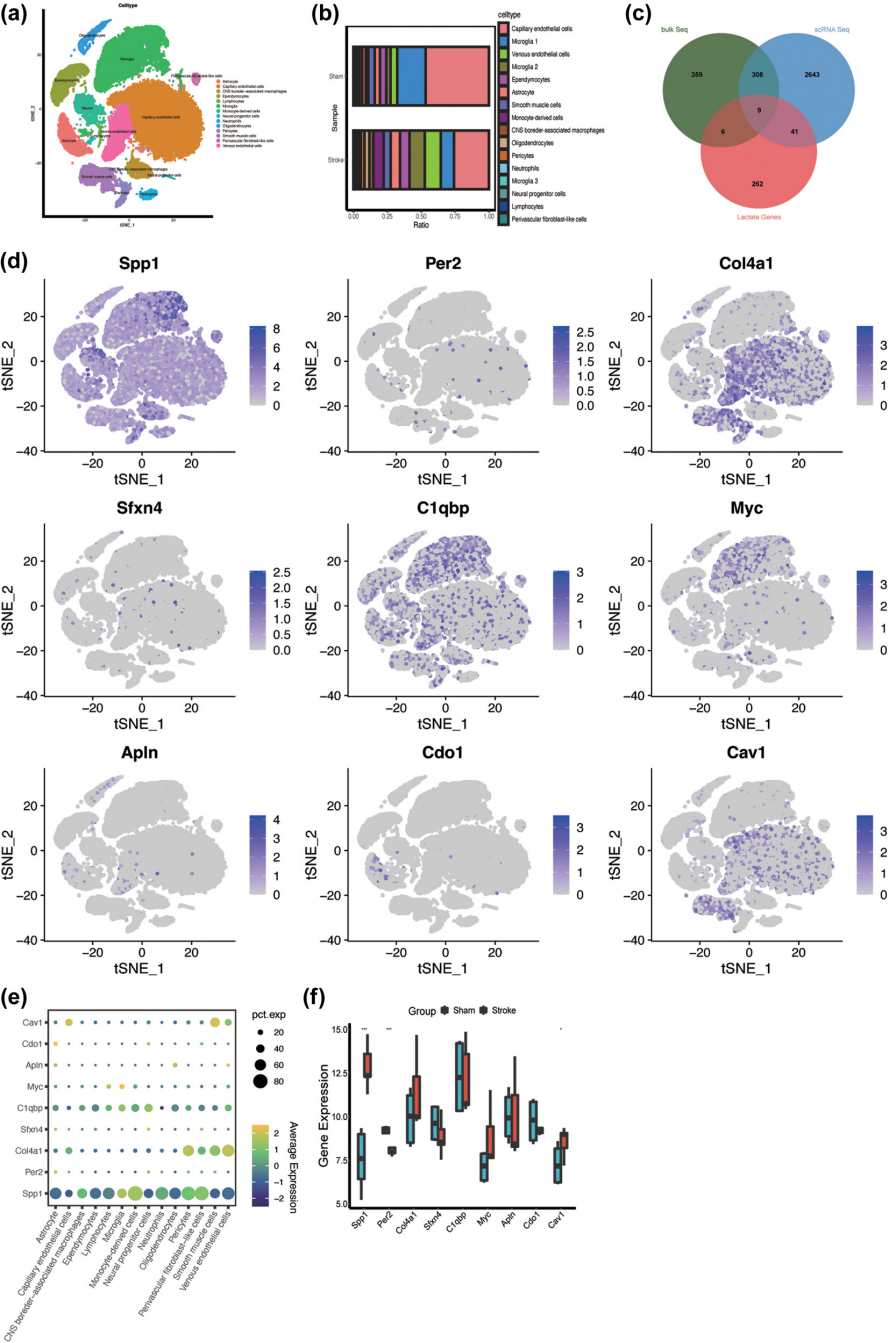
Cell subpopulation annotation of single-cell data and LR-DEG identification. (a) Cell annotation of 16 clusters, 16 clusters annotated into 14 cell types, astrocyte, capillary endothelial cells, CNS border-associated macrophages, ependymocytes, lymphocytes, microglia, monocyte-derived cells, neural progenitor cells, neutrophils, oligodendrocytes, pericytes, smooth muscle cells, perivascular fibroblast-like cells, and venous endothelial cells. (b) The cell ratio between sham MCAO groups. (c) Venn diagram displaying nine LR-DEGs in IS that overlapped bulk RNA sequencing analysis, single-cell RNA sequencing analysis, and LRGs. (d) and (e) The distribution and expression level of the nine LR-DEGs in cells; blue represents high expression in tSNE, and black represents low expression. The size of the circle represents the percentage it occupies. (f) The gene expression level of the nine LR-DEGs.

In the present study, we conducted a comprehensive scRNA-seq analysis to discern diverse cell populations within both the stroke and sham groups. The t-SNE plot shows the distribution and heterogeneity among the identified cell types (Figure 3a). Sixteen major cell types were annotated based on their specific marker genes and in accordance with previous studies [23], including astrocytes, capillary endothelial cells, CNS border-associated macrophages, ependymocytes, lymphocytes, microglia, monocytes, neural progenitor cells, oligodendrocytes, oligodendrocyte progenitor cells, and smooth muscle cells. The stacked bar plots (Figure 3b) depict the proportional changes in each cell type within the brains of the subjects who experienced IS. In the control group, capillary endothelial cells and microglial subset 1 were predominant, constituting >50% of the total cellular composition. In contrast, in the stroke groups, there was a significant upregulation in the expression of venous endothelial cells, microglia, and astrocytes. Subsequently, we performed differential expression analysis to compare the gene expression across each cell type within the two groups (Figure S1). Further intersecting these DEGs with bulk sequencing data, scRNA-seq results, and LRGs enabled us to successfully identify nine lactate-related differentially expressed genes (LR-DEGs) (Figure 3c). The t-SNE plot (Figure 3d) and bubble plot (Figure 3e) visualized the distribution and expression levels of the nine LR-DEGs across the 14 cell types. The box plot illustrates that in the stroke group, the expression levels of Spp1 and Cav1 were significantly elevated, whereas the expression level of Per2 was reduced (Figure 3f).

### 3.3 Intercellular communication analysis and signaling pathway analysis

**Figure 4:**
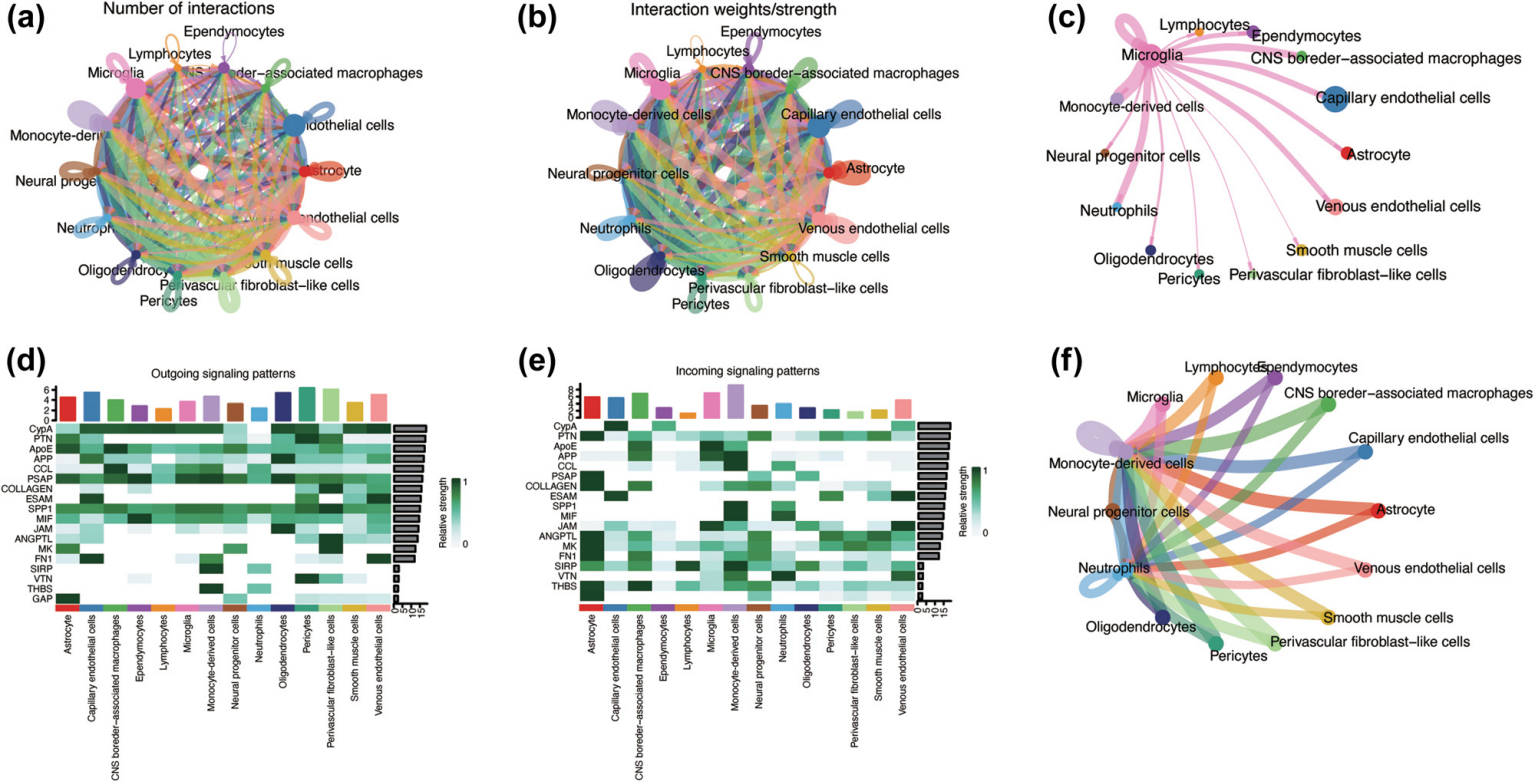
Intercellular communication analysis and signaling pathway analysis. (a) and (b) Circos diagrams illustrate the density of interactions between various pairs of cell types. The thickness of a line in the Circos plot corresponds to the intensity of interactions among distinct cell types. (c) Microglia communication with a diverse range of cell types. (d) and (e) The major signaling inputs and outputs among different cell types. (f) The circos diagram of Spp1 signaling pathway between different cell types.

To elucidate the intercellular relationships among the diverse cell types, we conducted an intercellular communication analysis. Figure 4a and 4b presents the cell–cell interaction network, with each node representing a distinct cell type. The edge interconnecting nodes denote the strength and frequency of interactions, with line thickness and density indicative of the extent of communication. Thicker lines imply more intense or frequent interactions. Notably, endothelial cells, microglia, and monocyte-derived cells occupy central positions within the network. Subsequently, we focused on intercellular communication between microglia and other cell types (Figure 4c). This analysis revealed significant interactions between microglia, monocyte-derived cells, and neutrophils, as evidenced by the robust edges connecting the corresponding nodes within the cell–cell interaction network. Figure S2 displays the overall communication conditions for all cell clusters in terms of quantity and strength. To reveal the biological functions associated with each cell type, we performed a comprehensive analysis of signaling pathways. The heatmap and circular plot (Figure 4d–f) illustrate the central role of microglia as primary signal emitters in the SPP1 pathway. Conversely, monocyte-derived cells have been identified as key signal recipients, underscoring their receptive roles in this biological interaction.

### 3.4 Microglia subcluster analysis

**Figure 5:**
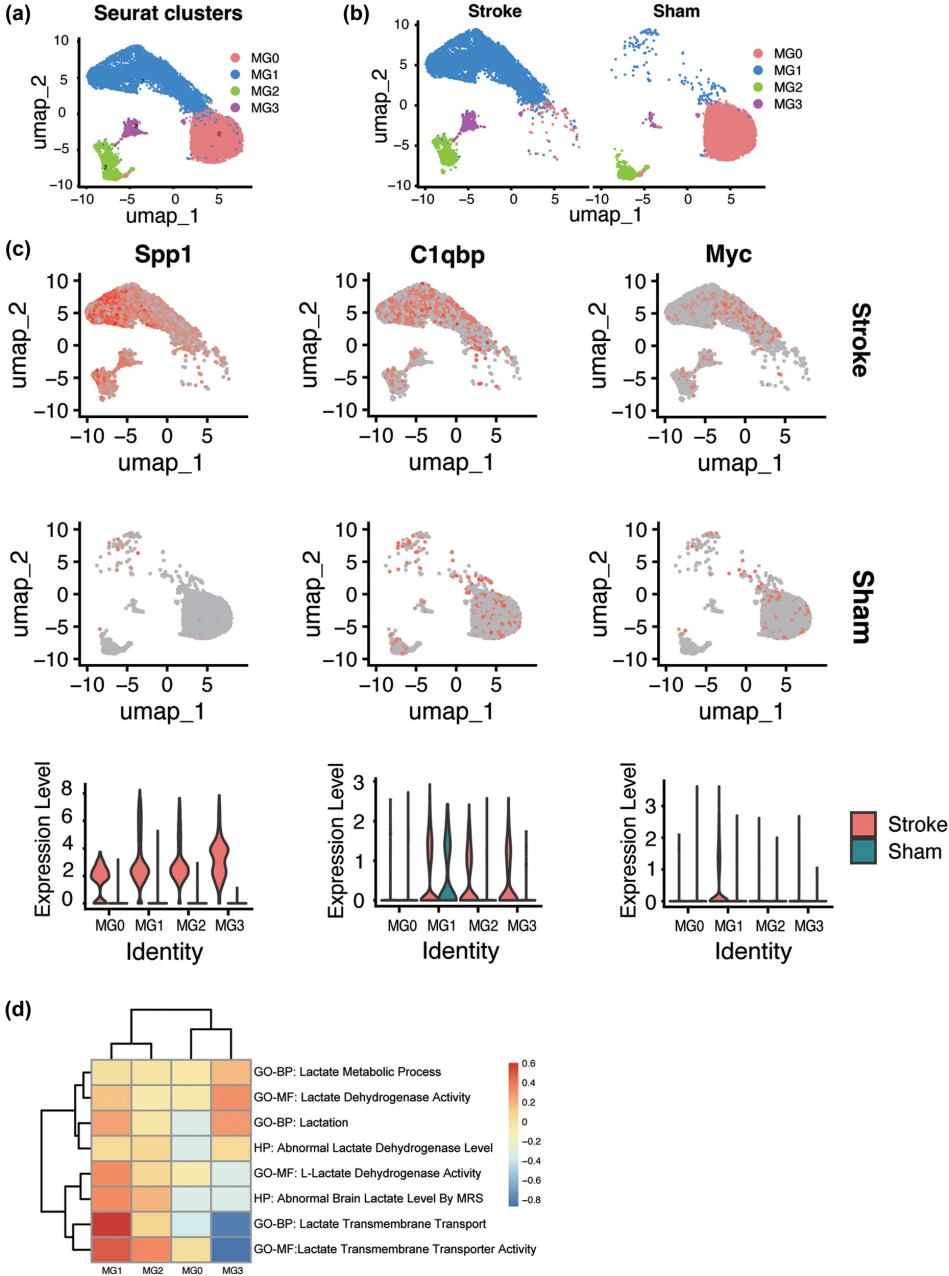
Microglia subclusters analysis and functional enrichment analysis of cluster 1. (a) The four subclusters of microglia. (b) Microglia density changes by groups. (c) Distribution of the three key LR-DEGs in microglia and the expression level of these genes. (d) GSVA analysis of four microglia subclusters.

To gain a comprehensive understanding of the regulatory functions of microglia at the onset of IS, we performed a subcluster analysis of the microglial population. Our findings demonstrated that microglia can be classified into four distinct subclusters (Figure 5a). In the sham group, MG0 was predominant, indicating that the majority of the cells were in a resting state. Conversely, in the MCAO group, MG1 was markedly increased and emerged as the predominant subcluster. This increase suggests the transition of these cells into an activated state, acting as effector cells that are critical for the pathogenesis of IS (Figure 5b). Additionally, the MCAO group showed elevated expression levels of Spp1, C1qbp, and Myc, which were primarily localized within MG1. Notably, C1qbp expression remained relatively stable across MG1 in both MCAO and sham groups 1-day post-stroke (Figure 5c). This observation implies that C1qbp may play a crucial role in the regulation of microglial resilience. Subsequently, we used the GSE225948 dataset to further explore the dynamic change of LRGs 14 days post-stroke. In this dataset, we observed a significant decrease in LRG expression levels at 14 days post-stroke compared to 1-day post-stroke, with C1qbp and Myc showing the most marked reductions, nearly returning to sham group levels (Figure S3c). Our results suggest that C1qbp and Myc are primarily active during the acute phase of IS. In contrast, the expression of Spp1 remains elevated in MG2 and MG3 at 14 days post-stroke, with levels similar to those on day 1 (Figure S3d–e). This indicates that Spp1 plays a key role in both the acute and chronic phases of IS. In summary, C1qbp and Myc are primarily involved in the acute response to IS, while Spp1 exerts neuroregulatory effects during both the acute and chronic phases of IS. GSVA analysis revealed that MG1 was highly enriched in lactate transmembrane transport and lactate transmembrane transporter activity, indicating that MG1 plays a vital role in lactate metabolism (Figure 5d).

### 3.5 Functional enrichment analysis of the microglia MG1 and GSEA analysis of LRGs

**Figure 6:**
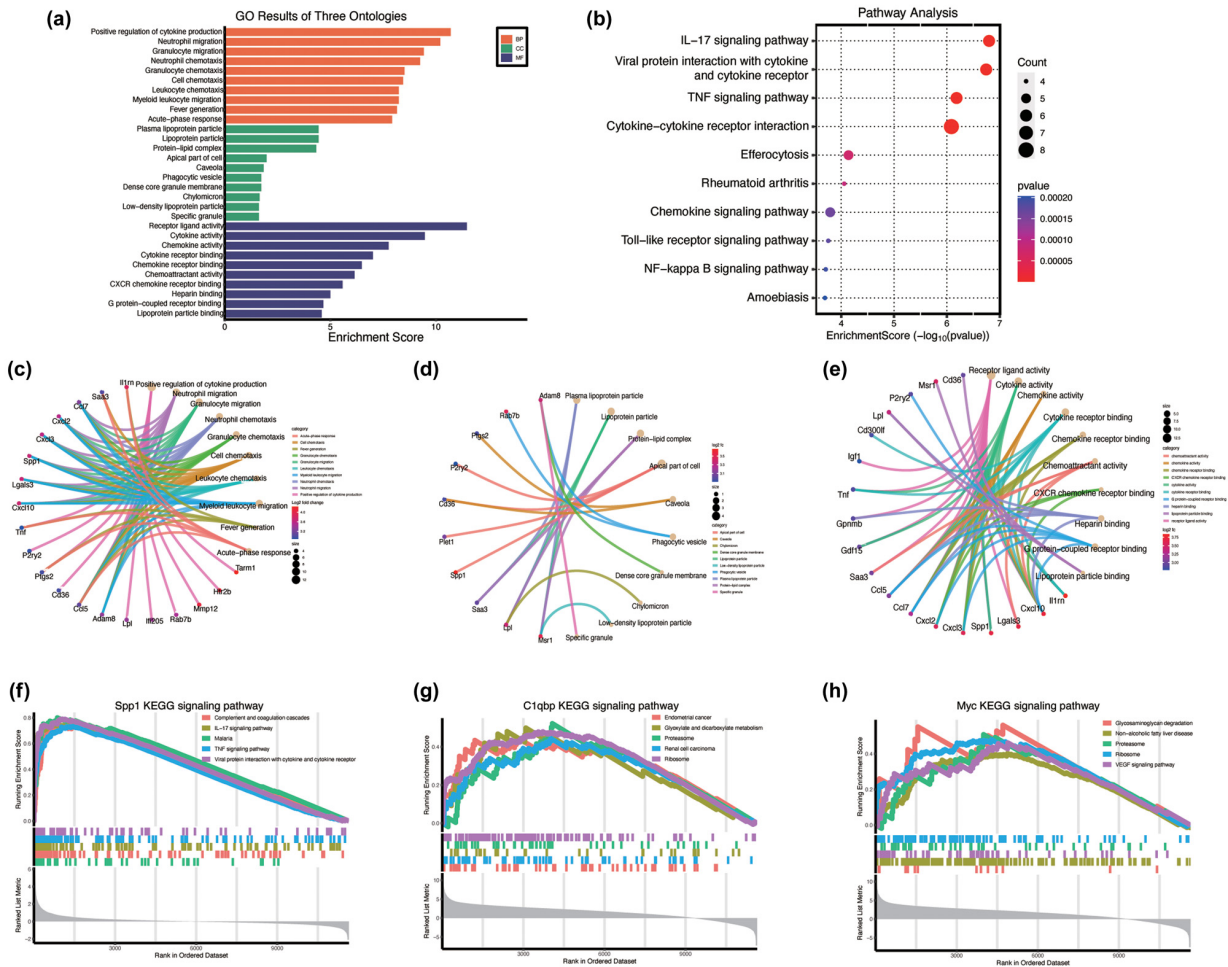
Functional enrichment analysis of the microglia MG1. (a) GO enrichment analysis of microglia MG1. (b) KEGG pathway enrichment analysis. (c)–(e). The chord diagram displays the connectivity between key genes and enriched GO terms. (f)–(h) GSEA of LRGs. KEGG signaling pathways involved in Spp1, C1qbp, and Myc.

GO (Figure 6a) and KEGG (Figure 6b) pathway analyses were performed to elucidate the biological functions specific to microglial MG1. Biological process (BP) analysis indicated that genes within this subcluster are primarily associated with “‘inflammatory response,” “immune response,” and “response to wounding,” highlighting the engagement of immune-related pathways. Furthermore, cellular component (CC) enrichment highlighted “plasma membrane” and “integral component of membrane,” suggesting a correlation between MG1 marker genes and membrane-associated structures. Molecular function (MF) enrichment emphasized “cytokine activity” and “receptor binding,” underscoring the critical role of cytokine signaling in mediating immune responses. KEGG pathway enrichment analysis further confirmed that pathways associated with immune responses and inflammation, particularly “cytokine–cytokine receptor interaction” and the “TNF signaling pathway,” were significantly enriched. This enrichment pattern suggests that microglial cells within MG1 are primarily involved in immune signaling and inflammatory processes. The chord diagram (Figure 6c–e) revealed the functional enrichment of the most highly expressed genes within MG1. The results revealed that Spp1 was significantly enriched across various functional processes, particularly in “immune cell migration,” “inflammation,” “acute-phase response,” and “cellular signal transduction.” GSEA of KEGG signaling pathways for the LRGs indicated that Spp1 is predominantly associated with the complement and coagulation cascades, IL-17 signaling pathway, and TNF signaling pathway. C1qbp was significantly enriched in endometrial cancer, glyoxylate and dicarboxylate metabolism, and proteasome pathways. Myc exhibited significant enrichment in glycosaminoglycan degradation, non-alcoholic fatty liver disease, and proteasome pathways (Figure 6f–h).

### 3.6 Pseudotime analysis of microglial subpopulations

**Figure 7:**
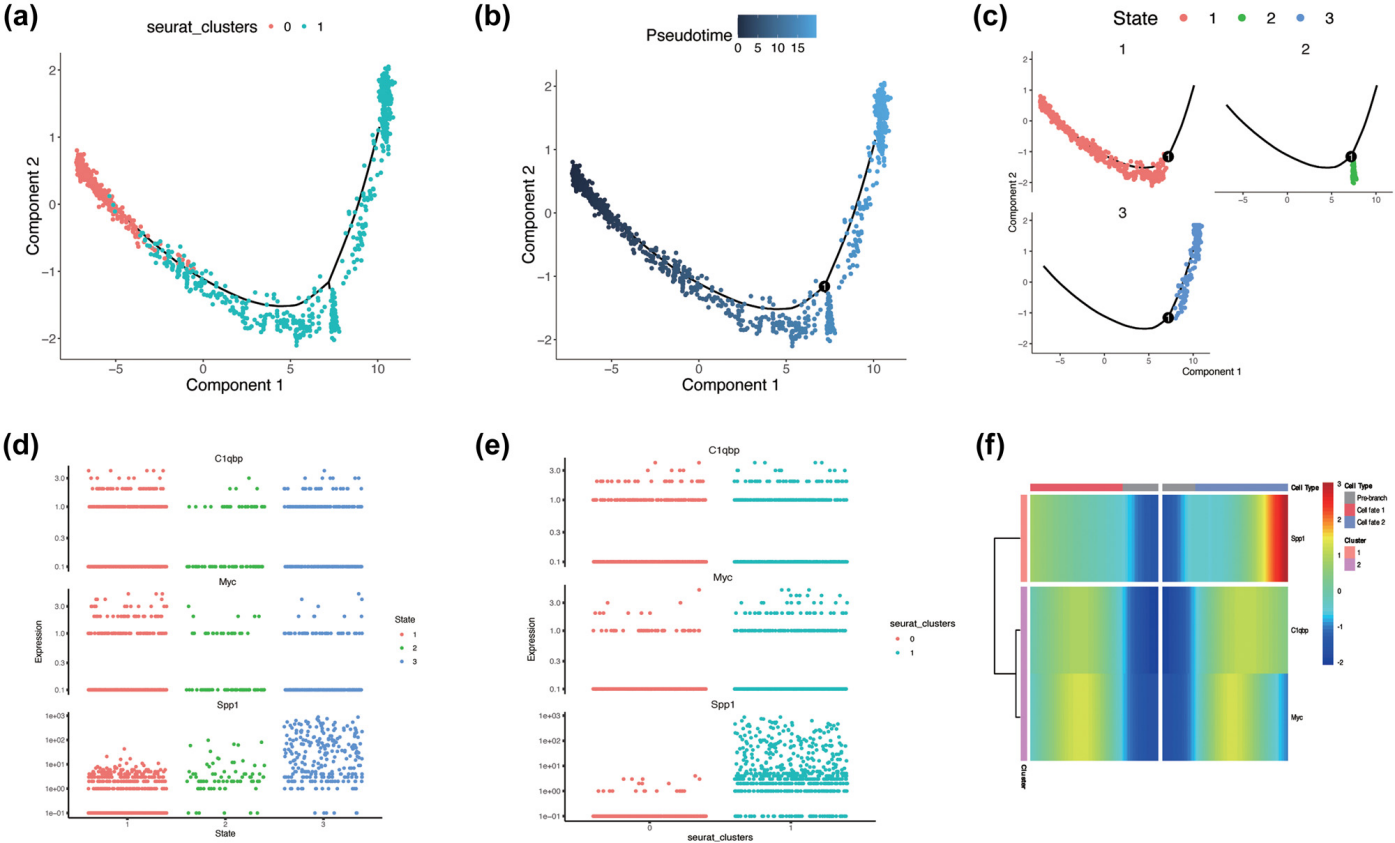
Single-cell trajectory analysis of microglia subclusters. (a)–(c). The three different differentiation states of microglia MG0 and MG1. (d) and (e) Dynamic expression of three key LR-DEGs across microglia states and subclusters. (f) Heatmap of LR-DEGs across different states.

Pseudotime analysis revealed that microglia subcluster 0 (MG0) and microglia subcluster 1 (MG1) exhibit distinct trajectories along two principal components, suggesting that these subpopulations may represent divergent cellular states. This observation is consistent with prior research, which documented the upregulation of MG1 following IS concurrent with the downregulation of MG0 (Figure 7a). The pseudotime values presented in Figure 7b illustrate a temporal progression from MG0 to MG1, where darker blue shades represent earlier stages, and lighter shades represent later stages. The U-shaped trajectory suggests a dynamic transition between cellular states. The microglial transition can be characterized by three distinct states (Figure 7c): red cells (state 1) occupy the early pseudotime, green cells (state 2) form a compact cluster at the midpoint of the trajectory, and blue cells (state 3) predominate in the later pseudotime stages. The expression levels of Spp1, assessed in the late stage (Figure 7d) and MG1 (Figure 7e), highlight its critical role in microglial responses to IS, particularly in later stages of inflammation and tissue repair. Furthermore, the heatmap (Figure 7f) shows that Spp1 expression is maximized at cell fate of 2.

### 3.7 Immune cell infiltration

**Figure 8:**
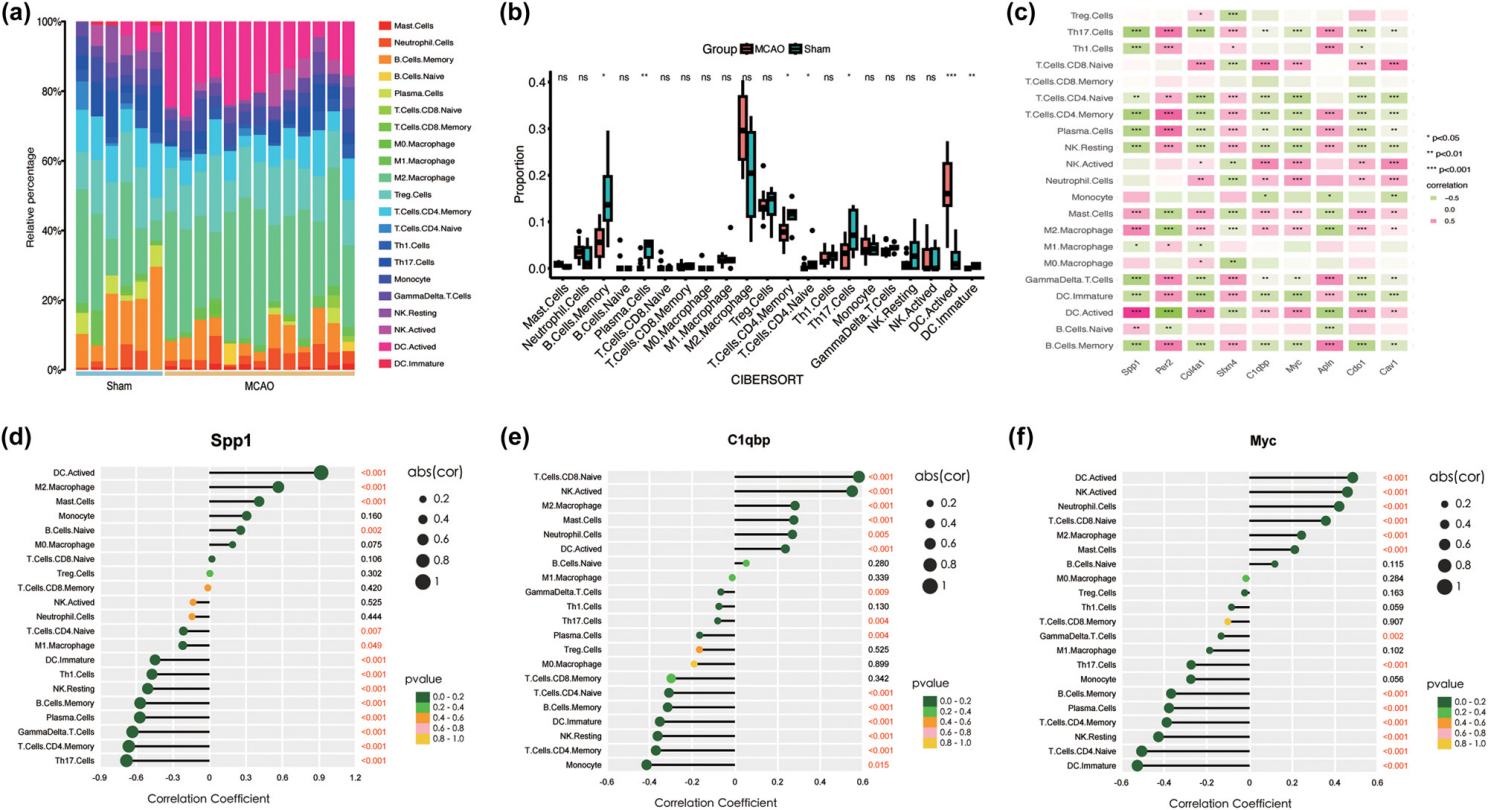
Immune cell infiltration. (a) Bar plot showing the composition of 21 types of immune cells across samples. (b) Correlation heatmap of 21 types of immune cells and LR-DEGs. Red indicates a positive correlation, and green indicates a negative correlation. *p-value <0.05, **p-value <0.01, and ***p-value <0.001. (c) Box plot of 21 types of immune cells across different samples. (d)–(f). Correlation between expression levels of the Spp1, C1qbp, and Myc. The larger the circle, the stronger the correlations.

To quantitatively evaluate the immune cell landscape after IS, we employed CIBERSORT on the bulk RNA sequencing data. The analysis revealed that macrophages were the predominant immune cells in the MCAO group (Figure 8a). In contrast, the sham group displayed a more uniform distribution of immune cells with lower activation markers, suggesting a baseline or quiescent state. Further analysis using a box plot revealed significant differences in the abundance of various immune cell types between the MCAO and sham groups (Figure 8b). Notably, T cells (CD8 memory), M2 macrophages, and plasma cells significantly increased in the MCAO group, whereas immature dendritic cells (DCs) and activated NK cells markedly decreased. The correlation heatmap (Figure 8c) underscored the strong association between LRGs and immune cell infiltration (Figure S4). Spp1 showed a strong positive correlation with activated DCs (activated), M2 macrophages, and mast cells and a negative correlation with Th17. Cells, T cells, CD4 memory, and gamma delta T cells (Figure 8d). C1qbp was positively correlated with T cells CD8, naive and activated natural killer cells (inactivated) and negatively correlated with monocytes, T cells, CD4 memory, and resting natural killer cells (NK, resting) (Figure 8e). Myc expression was positively associated with activated DC cells, activated NK cells, and a negative association with immature DC cells, naive CD4 T cells, and resting NK cells (Figure 8f). In summary, the observed changes in immune cell proportions following IS, in conjunction with gene correlation analysis, suggest that lactate metabolism plays a crucial role in modulating immune response. These observations offer valuable insights into the complex molecular mechanisms governing neuroinflammation and the subsequent post-stroke recovery.

## 4 Discussion

This study provides novel insights into the intricate relationship between lactate metabolism and microglial dynamics following IS. By integrating bulk sequencing and scRNA-seq, we systematically characterized the temporal expression trajectories of lactate-associated genes within microglia, highlighting dynamic changes in the expression levels of Spp1, C1qbp, and Myc during the late stages of post-ischemic recovery. These findings underscore the pivotal function of microglial lactate metabolism in the pathophysiology of stroke and offer promising therapeutic targets for modulating microglial responses in IS.

Lactate metabolism plays a crucial role in brain energy dynamics during and after an IS [28]. When oxygen levels are reduced due to ischemia, the brain shifts from oxidative phosphorylation to anaerobic glycolysis, leading to lactate accumulation [29]. Lactate, once considered merely a byproduct of anaerobic metabolism, has gained recognition as a significant metabolic substrate and signaling molecule that plays a crucial role in modulating immune and inflammatory responses in the brain [30]. Through our analysis, we identified nine LRGs that play a crucial role in modulating the reparative functions of various cell types in response to ischemic lesions. Specifically, Per2 and Cav1 have been shown to promote microglial polarization and inflammatory responses, which are essential for mitigating brain injury following ischemia [31,32]. Additionally, Col4a1, Apln, and Cav1 are significantly associated with the normal functioning of vascular endothelial cells and are critical for preserving the integrity of the blood–brain barrier (BBB). The aberrant expression of these genes may impair the recovery process after stroke [33,34]. Sfxn4 and Cdo1 are implicated in mitochondrial energy metabolism. We hypothesized that these genes may contribute to neuroprotection by enhancing mitochondrial biogenesis following IS [35,36]. Our findings revealed that these LRGs are predominantly expressed in monocyte-derived cells, astrocytes, microglia, and vascular endothelial cells, which are crucial for both health and disease. This distribution suggests that following IS, these genes contribute to maintaining cerebral homeostasis by preserving energy metabolism, antioxidant defenses, and anti-inflammatory processes.

Polarization of microglia is a critical process in the neuroinflammatory response following IS, and LRGs play a significant role in this process. Our results indicated that Spp1, C1qbp, and Myc are prominently enriched in microglial cells, which serve as the principal immune components of the CNS and are crucially involved in the pathogenesis of IS. The dynamic changes in the expression of these genes suggest their involvement in the transition of microglia from the MG0 microglia subcluster to the MG1 subcluster, underscoring the pivotal role of MG1 in the context of IS. Functional analysis of the MG1 subcluster revealed its crucial involvement in the immune response and anti-inflammatory activities. These cells play a significant role in modulating immune and inflammatory responses in the CNS post-ischemia. Therefore, we speculate that this transition is consistent with the polarization of microglia from a pro-inflammatory state to an anti-inflammatory state and that these three LRGs perform their neuroprotection at the late stage of IS.

Spp1, also known as osteopontin (OPN), is a multifunctional glycoprotein that is widely expressed in various tissues, including the CNS. Spp1 is significantly involved in modulating immune responses, inflammation, and tissue repair processes [37]. Post-IS, Spp1 expression is markedly upregulated in multiple cell types of the neurovascular unit, including microglia, endothelial cells, and astrocytes [38]. Elevated Spp1 expression plays a dual role: initially promoting a pro-inflammatory response aimed at clearing cellular debris and subsequently fostering a reparative environment that supports tissue healing [39]. Prior scRNA-seq studies have shown that certain subsets of microglia and macrophages exhibit increased Spp1 expression following ischemic events, which persist throughout the later stages of stroke recovery and play a critical role in mediating the transition from a pro-inflammatory to an anti-inflammatory microglial phenotype [40]. C1qbp is a multifunctional protein that plays a crucial role in diverse cellular processes and is primarily recognized for its regulatory function in the immune system. In addition to its immune functions, C1qbp is involved in energy metabolism by maintaining mitochondrial function, particularly in monocyte-derived cells and microglia. Within ischemic lesions, C1qbp upregulation enables microglia to meet increased metabolic demands and oxidative stress associated with neuroinflammation [41]. Myc is a key transcription factor that is essential for the regulation of cell cycle progression, apoptosis, and cellular transformation. In IS, Myc upregulation can mediate the transformation of microglia into dendritic-like cells, driven by the ERK/Myc signaling pathway. This pathway is crucial for microglial responses to ischemic injury, highlighting its role in post-stroke neuroinflammation [42]. Furthermore, it redirects metabolism towards oxidative phosphorylation under conditions of low glucose and high lactate levels, thereby promoting cell survival and function under hypoxic conditions by inhibiting glycolysis and increasing energy production [43]. Myc upregulation also promotes glycolysis and excessive lactic acid production by regulating the expression of GLUT1 and key glycolytic enzymes, including HK and PFK1 [44].

As the innate immune cell in the brain, microglia can exert its regulatory function in various diseases by interacting with other brain cells. In neurodegenerative disorders, microglia and neurons are interconnected primarily through the SPP1–ITGAV receptor–ligand pair, and this association is bidirectional. Additionally, microglia and astrocytes interact through the GAS6–MERTK and RELN–ITGB1 receptor–ligand pairs [45]. In an acute demyelination mouse model, activated astrocytes express multiple ligands, including Cx3cl1, Csf1, Il34, and Gas6, which act on both homeostatic and activated microglia, thereby potentially mediating microglial activation, recruitment, and enhancing their phagocytic activity [46,47]. It has also been reported that the Spp1 intercellular interaction pathway is significantly increased in mice with temporal lobe epilepsy. This interaction can be observed in all glial cells, with microglia and astrocytes displaying the strongest communication strength among others [48]. Gu et al. have also identified a microglial subcluster in rats with hemorrhagic stroke, characterized by the highest expression of Lcn2, Msr1, and Spp1 at 24 h post-stroke. These cells exhibit significant interactions with endothelial cells and participate in the inflammatory response. Furthermore, these microglia can also interact with neurons via the Lcn2-SLC22A17 signaling pathway to induce neuronal death [49]. In Alzheimer’s disease, specific transcription factors, such as MYC and CTNNB1, are altered in inhibitory neurons, leading to altered communication patterns between microglia and neurons. This microglia–neuron interaction may be mediated through the APOE–LRP8 ligand–receptor pair [50]. Although the analysis of cell–cell communication helps us understand the pattern of cellular interactions, it is, to some extent, unable to fully simulate the interconnections between cells in physiological and pathological conditions. First, when analyzing cell–cell communication, we use the point-to-point model of ligand–receptor to simulate cellular connections. However, under biological conditions, cellular connections are multi-dimensional, and relying solely on the ligand–receptor scale may not comprehensively reflect the strength of cellular interactions. Additionally, scRNA-seq analysis lacks spatial location information, and biological connections between cells often rely on spatial location. Moreover, scRNA-seq analysis lacks spatial location information, whereas biological connections between cells often rely on physical proximity. Although spatial transcriptomics can provide cell location information, it is difficult to accurately identify inter-cell interactions due to its low resolution. Cellular communication is a dynamic process, whereas scRNA-seq analysis primarily focuses on cellular changes at a specific point in time, failing to reflect changes in inter-cellular connections over time. Therefore, to achieve a comprehensive understanding of cellular interactions, we propose that the application of scRNA-seq analysis, combined with the verification of biological experiments, can provide solid evidence for understanding cellular communication.

Our findings indicate that C1qbp and Myc are predominantly expressed in the MG1 microglial subset post-ischemia and exhibit particularly high levels in the early stages of the disease, while in the chronic phase of IS, the expression level of these two genes is downregulated to the normal state. This observation suggests that C1qbp and Myc may serve as initiator genes in response to ischemic lesions, potentially inducing microglial polarization to counteract neuroinflammation. In contrast, Spp1 expression is upregulated at later stages of IS and will last for 2 weeks, suggesting a role for Spp1 in neurorestorative functions during the subsequent recovery phase following stroke.

Neuroinflammation following IS is triggered not only by resident immune cells but also by infiltrating immune cells from the peripheral immune system [51]. In our study, we characterized the post-IS infiltration patterns of immune cells and observed an increase in activated DCs and a concurrent decrease in memory B cells, plasma cells, memory CD4 T cells, and immature DCs. These findings highlight the dynamic and complex nature of the immune response after cerebral ischemia and demonstrate contrasting infiltration behaviors among specific immune cell populations. The elevated presence of activated DCs suggests a potential role in antigen presentation and the initiation of immune responses, while the diminished presence of other cell types, such as memory B cells and plasma cells, may indicate the resolution of initial inflammatory responses or a transition in the immune landscape towards a regulatory phenotype. These observations enhance our understanding of immune cell dynamics in the context of stroke and may inform the development of targeted immunomodulatory therapies.

In the present study, we employed an integrative approach of bulk-sequencing and scRNA-seq analyses to identify nine LRGs that exhibit dynamic expression patterns in microglia post-ischemia. By analyzing the distribution of these genes, we focused on temporal expression changes within microglia, potentially revealing the cellular response to IS. Additionally, by conducting a sub-clustering analysis of microglial populations, we delineated the phenotypic transitions of microglia at the onset of IS, thereby elucidating their biological functions throughout the disease process. However, our study has some limitations. The specific mechanisms underlying the action of these signature genes require further elucidation using both *in vitro* and *in vivo* experimental models.

## 5 Conclusion

In conclusion, through a series of bioinformatics analyses, we successfully identified nine signature genes (Spp1, Per2, Col4a1, Sfxn4, C1qbp, Myc, Apln, Cdo1, and Cav1) associated with IS and LRGs. Furthermore, three major LRGs are predominantly expressed in microglia and contribute to the polarization of these cells. Notably, the expression level of Spp1 increases significantly at the late stage of IS, suggesting that this gene may serve a neuroprotective function during later phases of the disease. Consequently, our findings provide novel insights for investigating dynamic alterations in LRGs within microglia. This discovery could facilitate the targeting of these LRGs at appropriate time points to modulate lactate metabolism, thereby potentially enhancing the therapeutic efficacy against IS.

## Supplementary Material

Supplementary Figure
