# Supplementary material for: Dynamic changes in lactate-related genes in microglia and their role in immune cell interactions after ischemic stroke
Source: Open Med (Wars). 2025 Apr 15;20(1):20251178. doi: 10.1515/med-2025-1178 (PMC12032981; doi:10.1515/med-2025-1178)
Supplement: Supplementary Figure [file med-2025-1178-sm.pdf]

Supplementary material

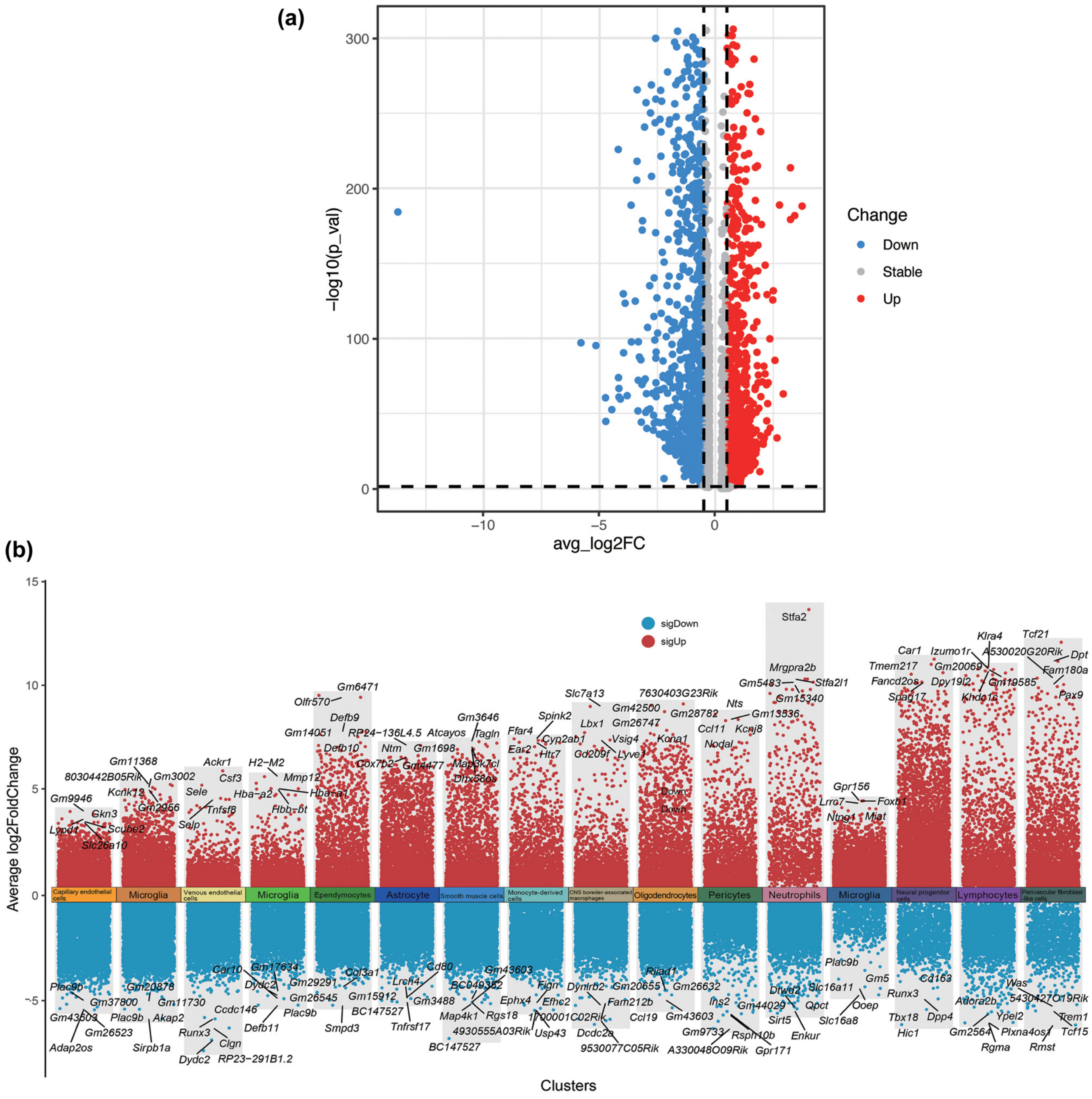

Figure S1: Differential expression analysis. (a) Volcano plot of the DEGs between MCAO and IS groups. (b) Volcano plot of the DEGs across MCAO and IS groups among in various cell types.

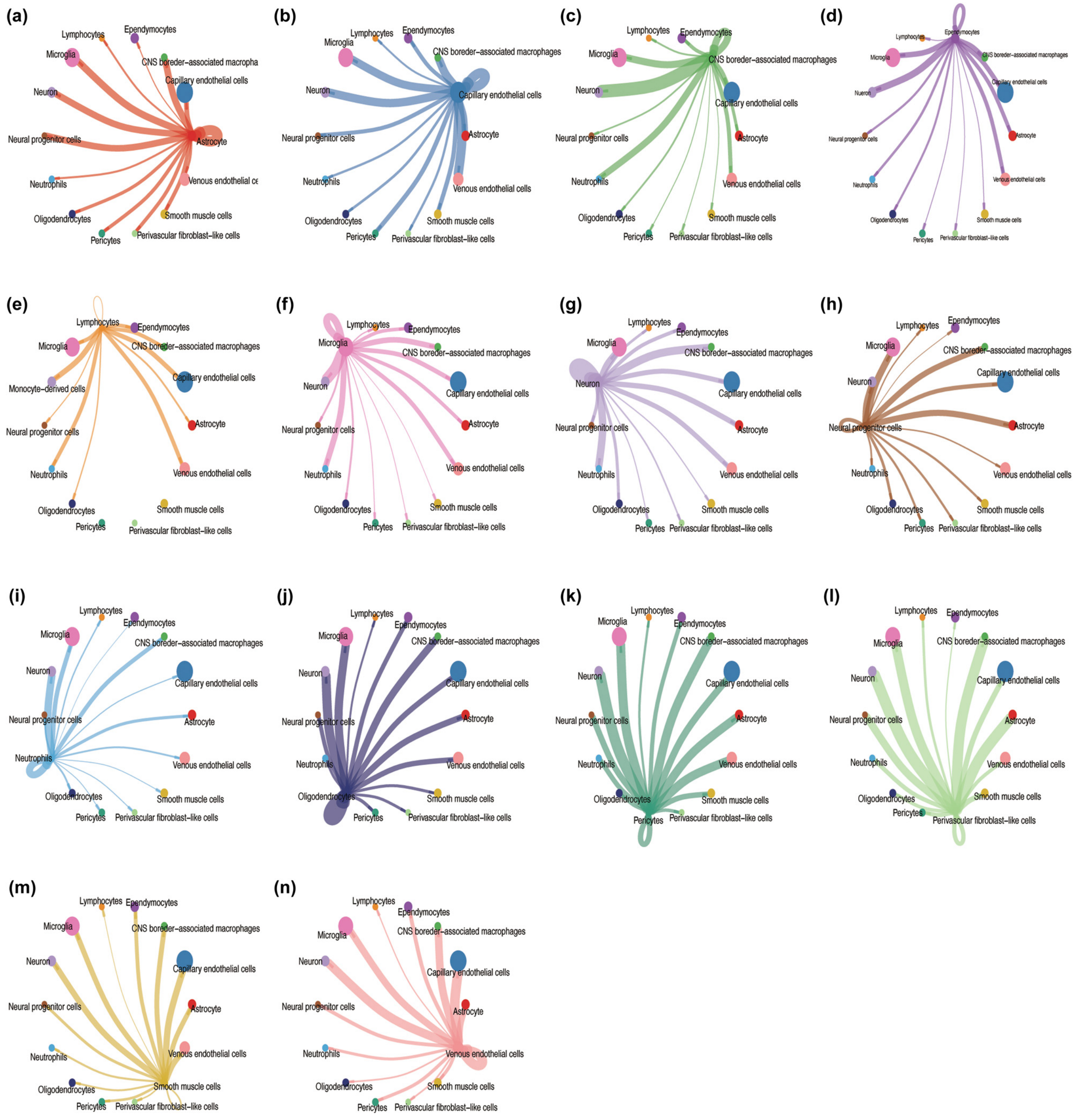

Figure S2: Intercellular communication analysis. (a–n) Cell-Cell communication among various cell type.

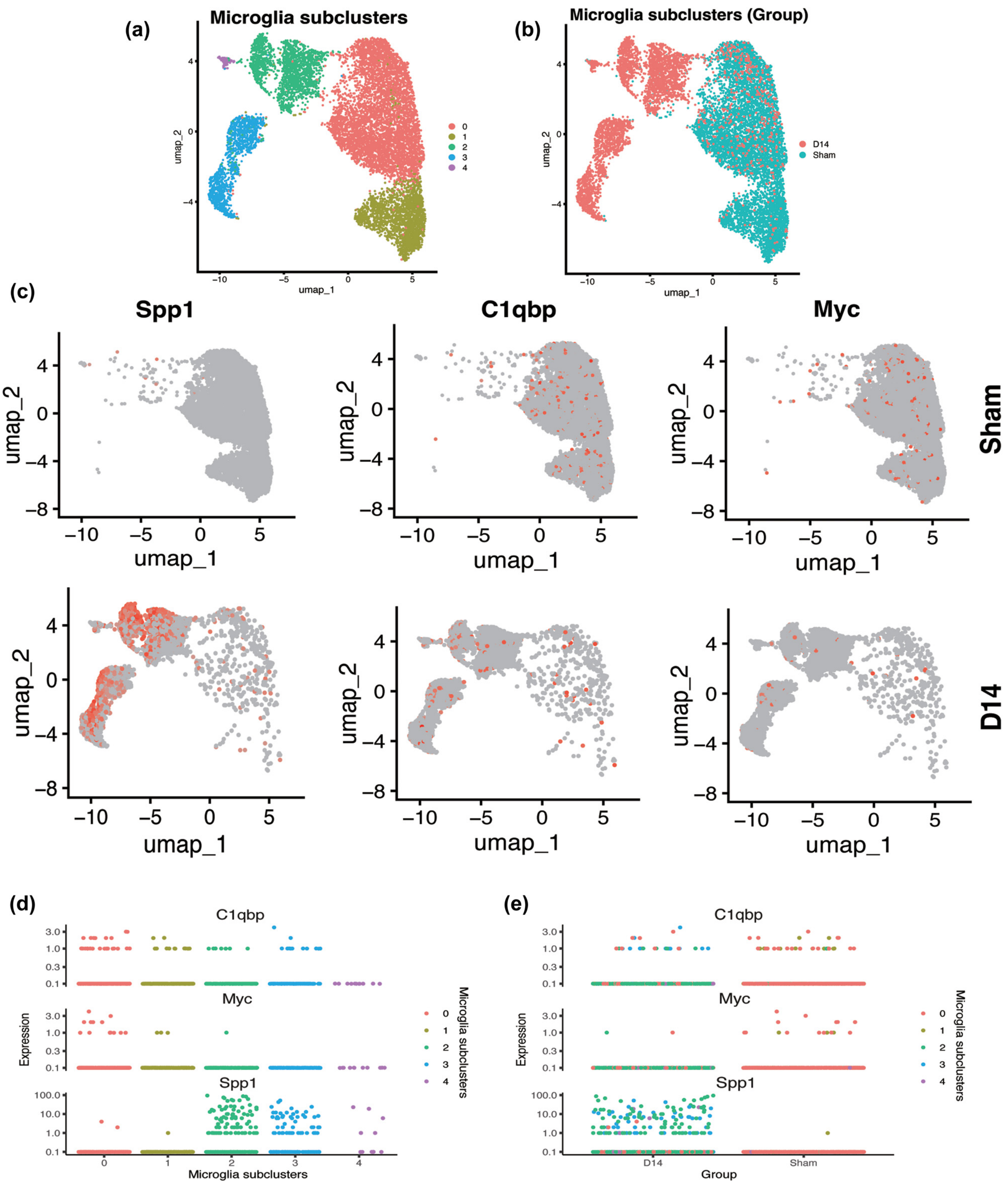

Figure S3: Analysis of microglia subclusters and dynamic changes of LRGs at 14 days post-stroke. (a) Microglia were classified into five distinct subclusters based on transcriptional profiles. (b) MG0 and MG1 were predominantly observed in the sham group, while MG2-MG4 were primarily identified at 14 days post-stroke. (c and d) Spatial distribution of LRGs across different microglia subclusters. (e) Comparative expression levels of LRGs in the sham group and at 14 days post-stroke (D14).

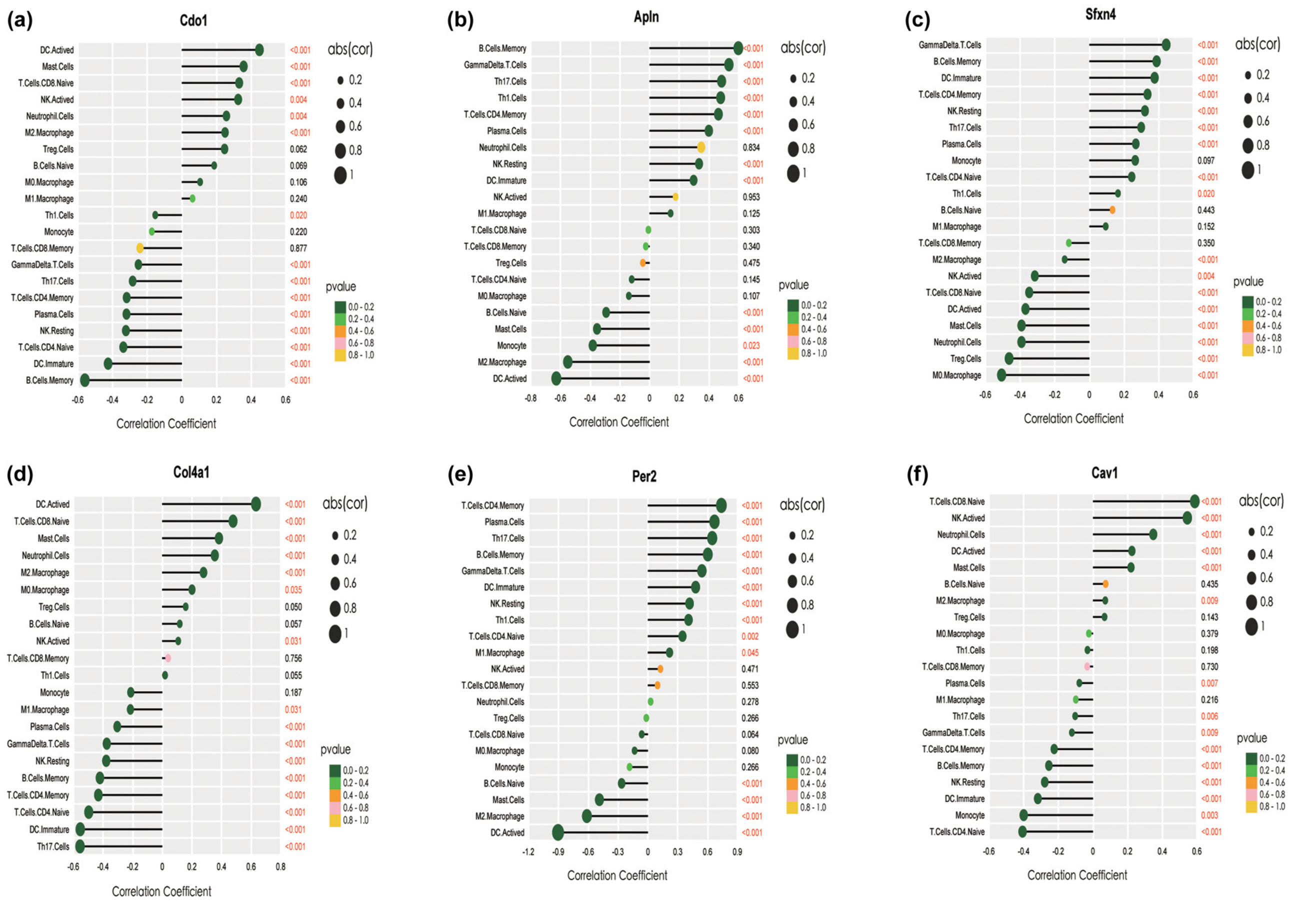

Figure S4: Immune cells infiltration. (a–f). Correlation between expression levels of the Cdo1, Apln, Sfnx4, Col4a1, Per2 and Cav1. The larger the circle, the stronger the correlations.
